# Supplementary material for: Impaired pattern separation in Tg2576 mice is associated with hyperexcitable dentate gyrus caused by Kv4.1 downregulation
Source: Mol Brain. 2021 Mar 30;14:62. doi: 10.1186/s13041-021-00774-x (PMC8011083; doi:10.1186/s13041-021-00774-x)
Supplement: Supplementary file 1 — Additional file 1: Figure S1. Comparison of the freezing levels in context A and B during contextual fear discrimination test. [file 13041_2021_774_MOESM1_ESM.pdf]

Supple 1

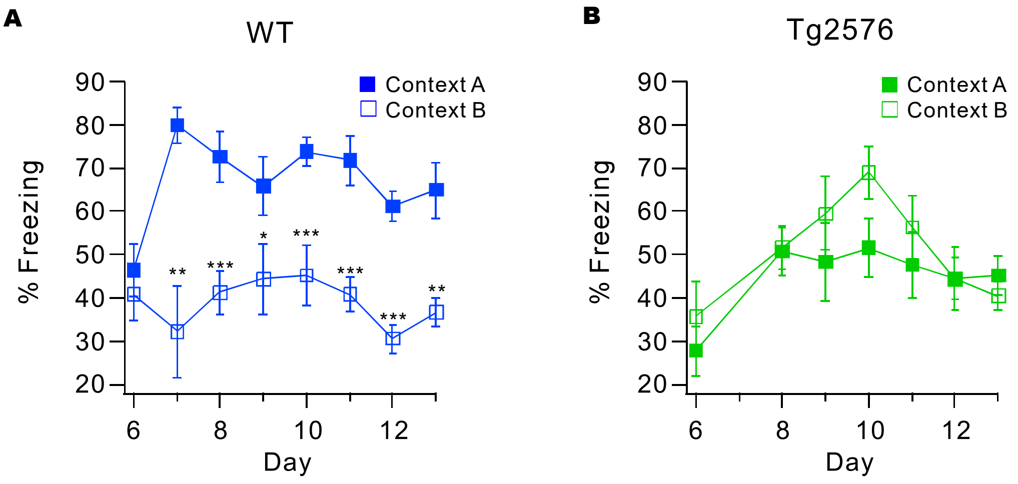
A
WT
90
80
70
60
50
40
30
20
% Freezing
Context A
Context B
**
***
*
***
***
***
**
6
8
10
12
Day
B
Tg2576
90
80
70
60
50
40
30
20
% Freezing
Context A
Context B
6
8
10
12
Day

Supplemental Figure 1. Comparison of the freezing levels in context A and B during contextual fear discrimination test. ***A***, Freezing level (%) of WT mice in context A (filled square) and context B (open square) from day 6 to day 13. ***B***, Freezing level (%) of Tg2576 mice in context A (filled square) and context B (open square) from day 6 to day 13. Mean ± S.E.M., *, $P < 0.05$, **, $P < 0.01$, ***, $P < 0.001$. Without star, $P > 0.05$.
